# Environmental microbial diversity and water pollution characteristics resulted from 150 km coastline in Quanzhou Bay offshore area

**DOI:** 10.3389/fmicb.2024.1438133

**Published:** 2024-07-04

**Authors:** Siqi Ding, Jiamin Chang, Wenzhou Zhang, Shouping Ji, Yulang Chi

**Affiliations:** ^1^College of Oceanology and Food Science, Quanzhou Normal University, Quanzhou, China; ^2^School of Pharmacy, Quanzhou Medical College, Quanzhou, China; ^3^Fujian Province Key Laboratory for the Development of Bioactive Material from Marine Algae, Quanzhou Normal University, Quanzhou, China

**Keywords:** offshore area, sediment, environmental microbiology, water quality, Quanzhou Bay

## Abstract

As a typical transitional area between the land and sea, the offshore area is subjected to the triple synergistic pressure from the ocean, land, and atmosphere at the same time, and has obvious characteristics such as complex and diverse chemical, physical, and biological processes, coupled and changeable environmental factors, and sensitive and fragile ecological environment. With the deepening of the urbanization process, the offshore area has gradually become the final receptions of pollutants produced by industry, agriculture, and service industries, and plays a key role in the global environmental geochemical cycle of pollutants. In this study, the Quanzhou Bay offshore area was selected as the research object. Sediment and water samples were collected from 8 sampling points within about 150 km of coastline in the Quanzhou Bay offshore area. 16s rDNA high-throughput sequencing method was used to investigate the variation rule of microbial diversity in the offshore area, and multi-parameter water quality analysis was carried out at the same time. The results showed that the distribution characteristics of microbial communities and water quality in the Quanzhou Bay offshore area showed significant differences in different latitudes and longitudes. This difference is closely related to the complexity of offshore area. This study can provide scientific support for protecting and improving the ecological environment of offshore areas.

## 1 Introduction

The offshore area is the zone where the interaction between the land and the ocean is the strongest, with rich biodiversity and high productivity. It is of great value in pollution degradation, climate regulation, and many other aspects (Crain et al., 2009; Li et al., 2010). With the deepening of urbanization, the offshore area has gradually become the final reception of pollutants produced by industry, agriculture, and service industries (Zhai et al., 2020). There are a total of 9,600 land-based sources of pollution in the sea in China, including more than 740 rivers entering the sea, more than 7,500 sewage outlets entering the sea, and more than 1,350 flood discharge outlets. It also plays a key role in the global environmental geochemical cycle of pollutants (Li et al., 2015). In order to protect and improve the ecological environment of offshore areas, China promulgated the Management Measures for Coastal Environmental Functional Zones to strengthen the management of coastal environmental functional zones (Cao and Wong, 2007). However, the composition of pollutants in the offshore area is complex and diversified, and the overall pollution is still relatively serious, and the trend of ecosystem degradation has not been fundamentally reversed (Dai et al., 2022). As stated in the ecological and environmental issues and policies for the sustainable development of China’s oceans, the environment of China’s offshore area is in a situation of composite pollution, and land-based pollution is serious, which is difficult to prevent and control. Due to the special geographical characteristics of the offshore area, it has become a special area of complex pollution. At the same time, it also makes it an excellent place to carry out ecotoxicological tests. Due to the synergistic effect of many environmental factors, it is urgent to evaluate the ecological environment of the offshore area (Beyer et al., 2014; Gissi et al., 2021). Regarding the offshore area, under the influence of tidal phenomena, the exchange of water bodies is frequent, and the environment of surface sediments is complex and changeable (Liu et al., 2023). Coastal microorganisms are an important part of the marine biosphere and play a key role in the whole marine environment (Oberbeckmann and Labrenz, 2020). It is not only an important driving force of the marine biogeochemical cycle and the key point of marine pollution control, but also closely related to human health, the development of the mariculture industry, and the security of marine ecosystems (Elgendy et al., 2023). It is of great significance for protecting the marine environment, protecting human health, and promoting the sustainable development of the marine economy to carry out systematic research on environmental microorganisms in offshore areas, clarify the change law of microbial diversity, and carry out characteristic microbial monitoring (Short et al., 2021; Wang et al., 2022; Olabi et al., 2023). Revealing the structure, abundance and key driving factors of microbial communities in the offshore area can improve the ability of pollution control in offshore areas (Chen et al., 2019).

The offshore area of Quanzhou Bay is an ecological environment rich in a variety of microorganisms, including actinomycetes, bacteria, algae, etc. In the study of ecosystem diversity and function and the interaction between microorganisms and the environment, more than 1000 strains of bacteria were isolated and cultured from the sediments of three typical habitats in Quanzhou Bay, including more than 390 genotypes. A total of 138 strains of actinomycetes were isolated through four media, of which 4% were potential new species or new genera. A total of 138 actinomycetes were isolated from 6 orders, 24 genera, and 62 genotypes, including a new species, Maribellussediminis, which is a facultor anaerobes with nitrogen-fixing ability and has a nitrogen-fixing gene cluster on its genome. In addition, the comparison of 62 genotypes with the bacterial taxa obtained by MiSeq16S amplicons showed that the microbial resources in the coastal sediments of Quanzhou Bay were vibrant (Huang et al., 2020b,^2021^). These studies provide a new research direction for the interaction between microorganisms and the environment in the coastal waters of Quanzhou Bay. In the study of the relationship between microbial resources and human health, a new kind of red Marine bacteria, Spartinivicinusruber, was found in Quanzhou Bay. Two red compounds, heptylprodigiosin and cycloheptylprodigiosin, were simultaneously isolated by strain culture, compound extraction, separation, mass spectrometry, and nuclear magnetic resonance spectroscopy. A bacteriostatic experiment showed that the Spartinivicinusruber S2-4-1 h bacterium’s genome and gene annotation analysis found that the two red pigment syntheses are formed by a gene containing 29 gene clusters (Huang et al., 2020a). In summary, the current status of microbial research in the coastal waters of Quanzhou Bay is characterized by multi-discipline and multi-level depth, which provides an important scientific basis for us to understand the marine microbial ecosystem further, develop and utilize microbial resources, and study the relationship between microorganisms and the environment and human health.

Water quality is an important element affecting ecological health and sustainable development (Cheng et al., 2022). Its quality not only directly affects the quality of human life, but also profoundly affects the diversity of microbial communities, the stability of ecosystems, and their resilience (Lin et al., 2022). However, with the acceleration of industrialization and the improvement of urbanization, the problem of water pollution has become increasingly prominent, which has brought huge pressure to the ecological environment (Ma et al., 2022). Therefore, it is necessary to deeply understand the close relationship between water quality and the environment, understand the significant impact of water quality on human health, and establish effective environmental protection strategies (Cheng et al., 2022; Lin et al., 2022).

The research area of this experiment is Quanzhou Bay offshore area. Quanzhou Bay is located in the southeast coastal area of Fujian Province, China, at the junction of the Jinjiang River and Luoyang River, with rich water resources and a good ecological environment, is one of the key protected ecosystems in China (Chen et al., 2016). In addition, Quanzhou Bay, as one of the important port cities on the southeast coast of China, the water quality of its coastal waters is directly related to the quality of life of residents and the sustainable development of the economy (Chen et al., 2016). According to the survey, although the ecological environment in the region is generally stable, it is still significantly affected by human activities (Li et al., 2010). Therefore, how to improve the water quality of the coastal area of Quanzhou Bay has become an urgent problem to be solved (Yu et al., 2008; Wang et al., 2022).

To understand the ecological environment status and influencing factors in the offshore area of Quanzhou Bay, the water quality and sediment microorganisms were investigated by *in situ* sampling. The effects of environmental factors on microorganisms and water quality and the relationship between microbial community and environmental factors were analyzed and summarized. It provides the basic data for further understanding the impact of microbial community characteristics and water quality changes on the overall ecological environment in the coastal waters of Quanzhou Bay and provides the basic basis for the protection measures of the coastal waters.

## 2 Materials and methods

### 2.1 Sampling points selection and sample collection

**FIGURE 1:**
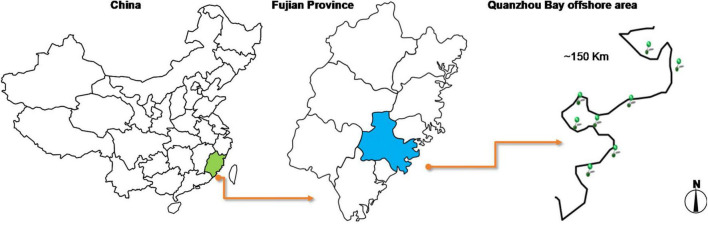
Sampling sites in the offshore area of Quanzhou Bay.

Quanzhou Bay is the most important of the three bays (Quanzhou Bay, Weitou Bay, and Shenzhen-Shanghai Bay) in Quanzhou, located on the southeast coast of Fujian Province. It is a semi-closed bay that is joined by Jinjiang River and Luoyang River into the sea, with a water area of 500 km^2^ (Yu et al., 2008). Based on the investigation of the geographical location of Quanzhou Bay, the sampling points in Hongtawan park (HTW), Xiangzhi park (XZP), Shishi Liusheng tower (LST), Shishi wetland (SSS), Binhai park (BHP), Quanzhou Oulebao Ocean Kingdom Park (OLB), Qingshan Bay Tourist area (QSW) and Dongdi countryside (DDC) were selected as the sampling points (see Figure 1 and Table 1 for the geographical information of each sampling point). And all the sampling sites are about 500 m from the land. The water quality index of each sampling point was measured on the spot. In addition, the sediment was collected by the principle of random, equal amounts and multi-point mixing, which was put into the ice box after collection and sent to the laboratory within 2 h for testing.

**TABLE 1:** Latitude and longitude of the sampling points.

Sampling points	Longitude	Latitude
HTW	118°72′	24°69′
XZP	118°78′	24°70′
LST	118°73′	24°81′
SSS	118°68′	24°80′
BHP	118°66′	24°85′
OLB	118°75′	24°86′
QSW	118°88′	24°90′
DDC	118°90′	24°94′

HTW, Hongtawan park; XZP, Xiangzhi park; LST, Shishi Liusheng tower; SSS, Shishi wetland; BHP, Binhai park; OLB, Quanzhou Oulebao Ocean Kingdom Park; QSW, Qingshan Bay Tourist area; DDC, Dongdi countryside.

### 2.2 Measurement of water quality indexes

The pH, dissolved oxygen (LDO), conductivity (CDC), temperature (TEM), salinity (SAL), NO_3_^–^ and NH_4_^+^ of water quality were determined by the HQ40d dual-channel multi-parameter water quality analyzer (Hach Company, Loveland, CO, United States). *In situ* water quality determination was performed. Each site was measured three times, and the measured data were recorded and plotted against their mean values.

### 2.3 DNA extraction and PCR amplification

The DNA contained in the samples was extracted by CTAB detection, and its purity and concentration were detected by agarose gel electrophoresis with 1% concentration (Zhang et al., 2024). In addition, an appropriate amount of sample is weighed and placed in a centrifuge tube and diluted to 1 ng/μL with sterile water. PCR amplification of V3 to V4 variable regions was performed using primer 341F (5′-CCTAYGGGRBGCASCAG-3′) and 806R (5′-GGACTACNNGGGTATCTAAT-3′). The PCR amplification reaction process is first heated at 95°C for 5 min to denature the first DNA of the template, followed by 30 cycles at 98°C (10 s), 50°C (30s), 72°C (30 s), and finally held at 72°C for 5 min and amplified with the above primers (Minas et al., 2011).

### 2.4 Library generation and 16s rDNA sequencing

The NEB Next ^®^ Ultra DNA Library Prep Kit was used to construct the library, and Agilent 5400 and Q-PCR were used to detect and quantify the constructed library. After the library was qualified, the Illumina sequencing platform was used for on-computer sequencing.

### 2.5 Statistical analysis

In this experiment, the software used for data processing and plotting is IBM SPSS Statistics 20 (International Business Machines Corporation, Armonk, New York) data editor and GraphPad Prism 8 (GraphPad, La Jolla, California, USA). In addition, the Wekemo Bioincloud platform was also used to conduct data analysis.^1^ When sequencing was completed, all data were expressed as a mean ± SD, and the independent sample *t*-test was used to estimate sample differences between different sampling points. Differences were considered to be statistically significant when *p* < 0.05 (*p < 0.05,**p < 0.01,***p < 0.001).

## 3 Results

### 3.1 Influence of latitude and longitude on microorganisms

**FIGURE 2:**
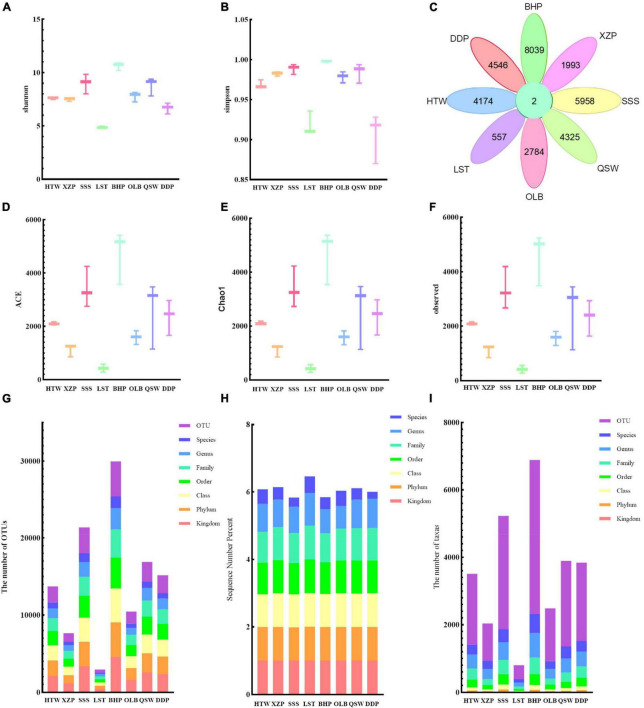
Alpha diversity and species notes. **(A)** Shannon. **(B)** Simpson. **(C)** Shared and common features across the group are displayed in the Petal diagram. **(D)** ACE. **(E)** Chao1. **(F)** Observed features. **(G)** The number of assigned OUTs. **(H)** The sequence number percent. **(I)** The number of taxas.

Data at different latitudes and longitudes were processed and the results of the α-diversity indexes were analyzed. As shown in Figure 2, the α-diversity indexes including Shannon (Figure 2A), Simpson (Figure 2B), ACE (Figure 2D), Chao1 (Figure 2E), and observed species characteristics (Figure 2F) were analyzed. The species diversity and community diversity of microorganisms varied with different latitudes and longitudes. With the increase of latitude, the observed species characteristics, ACE and Chao1 had the same change rule. As a whole, the values of ACE and Chao1 were increased with the increase of latitude. However, the species diversity decreased with the increase of latitude. Moreover, it could be seen from the results that the longitude and latitude of the maximum mean of Shannon, Simpson, ACE, Chao1, and the observed species features were all 118° 66′E; 24° 85′N, while the latitude and longitude of the minimum mean were all 118° 73′E; 24° 81′N. As for Shannon’s and Simpson’s values alone, there is no obvious change rule with the increase in latitude. In addition, after calculating the characteristics of unique and common to each sample group based on the given abundance table, the number of operational taxa (OTUs) shared by the dataset is 2, while 8,039 are unique to BHP, 557 are unique to LST, and so on (Figure 2C). At different species grading levels, the proportion of OTU (Figure 2G) and the number of taxas (Figure 2I) show the basic information of species annotation statistics, and it can be seen that the species annotation of BHP is better. And at different species grading levels, the percentage of sequence number (Figure 2H) shows the basic information of species annotation statistics, and it can be seen that the species annotation of LST is better.

**FIGURE 3:**
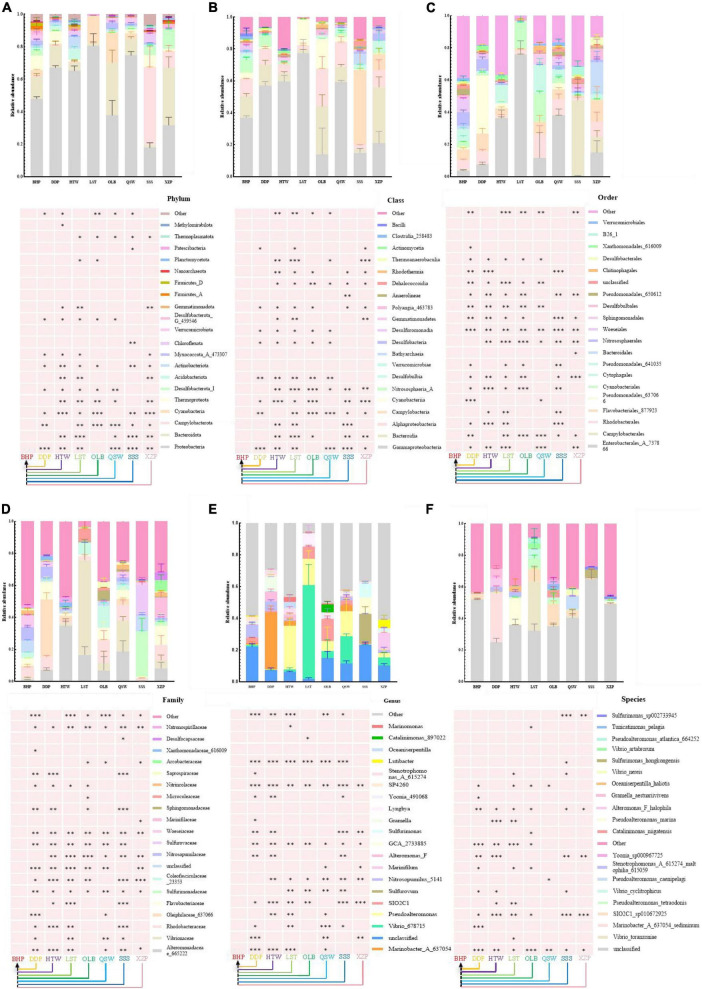
Sediment microbiota composition at different taxonomic ranks which are **(A)** Phylum. **(B)** Class. **(C)** Order. **(D)** Family. **(E)** Genus. **(F)** Species. *p < 0.05, **p < 0.01, ***p < 0.001.

As shown in Figure 3, it can be seen that the composition of the microbial community changes with the changes in latitude and longitude. In this present work, different sediment microorganisms in the coastal waters of Quanzhou Bay were measured, and the changes in microbial community composition under different species classifications (phylum, class, order, family, genus, and species) were analyzed. At the phylum level (Figure 3A), the results showed that the top 5 microbial communities in the sediment were mainly Proteobacteria, Bacteroideta, Campylobacterta, Cyanobacteria, and Thermoproteota. Compared with BHP as a reference, with the decrease of latitude, Cyanobacteria and Proteobacteria decreased significantly (*p* < 0.01). With the increase of latitude, Campylobacterta decreased significantly (*p* < 0.01), and Desulfobacterota_I increased significantly (*p* < 0.05). At the class level, also using BHP as a reference, the relative abundance value of Cyanobacteria increased regardless of whether the latitude increased or decreased (*p* < 0.01), and the relative abundance of Cyanobacteria was the highest among the top 5 microbial communities (i.e., Gammaproteobacteria, Bacteroidia, Alphaproteobacteria, Campylobacteria and Cyanobacteria) (Figure 3B). This is because the temperature mainly affects the growth of Cyanobacteria, most Cyanobacteria can grow at 10–40°C, and the temperature in the coastal waters of Quanzhou varies between 14 and 22°C. The changes in sediment microbial community composition in other species classification levels (i.e., order, family, genus, and species) are summarized below. At the order levels, the relative abundance of Woeseiales was the highest (*p* < 0.01). Woeseiales is a model strain (new species) produced in China, its destructive disturbance to coastal wetlands will lead to significant differences in the composition and abundance of bacterial communities (Figure 3C). At the family level, the highest species in HTW, LST and SSS were Alteromonadaceae_665222, VIbrionaceae, and Sulfurimonadaceae, respectively (Figure 3D). At the genus level, Lutibacter, SIO2C1, SP4240 with the significance level increased regardless of latitude increase or decrease (*p* < 0.01) (Figure 3E). In addition, at the species level, the relative abundance of *Alteromonas_F_halophila* was generally small (*p* < 0.05), but larger at the DDP position (*p* < 0.01) (Figure 3F). The above results indicate that the species of sediment microbial communities at different species scales vary greatly when the latitude and longitude are different. It can be seen that the types of microbial communities are affected by latitude and longitude, and are mainly affected by latitude.

**FIGURE 4:**
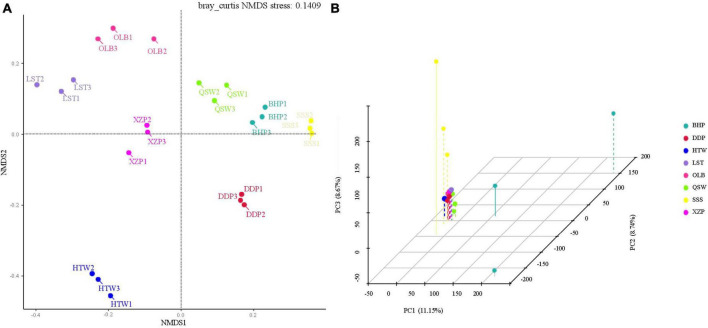
Environmental microorganism beta diversity analyses. **(A)** Non-metric multidimensional scaling (NMDS). **(B)** Principal component analysis (PCA).

The present work also performed non-metric multidimensional scaling (NMDS) and principal component analysis (PCA) for the β-diversity analysis of sediment microbial communities. It can be seen from the NMDS diagram that in the sediment microbial community, the sample points of XZP, HTW, LST, and OLB gather on the left side of the NMDS1 axis, indicating that their community structure is similar, which indicates that when there is little difference between the longitude, the structure of the sediment microbial community is similar. The sample points of SSS, BHP, QSW, and DDP were concentrated on the right side of the NMDS1 axis, indicating that their community structure was significantly different. It shows that the structure of sediment microorganisms was significantly different when the longitude difference was obvious (Figure 4A). In addition, this conclusion can also be reached in the three-dimensional space diagram of the PCA of β diversity (Figure 4B).

### 3.2 Analysis of characteristic species between different sites

**FIGURE 5:**
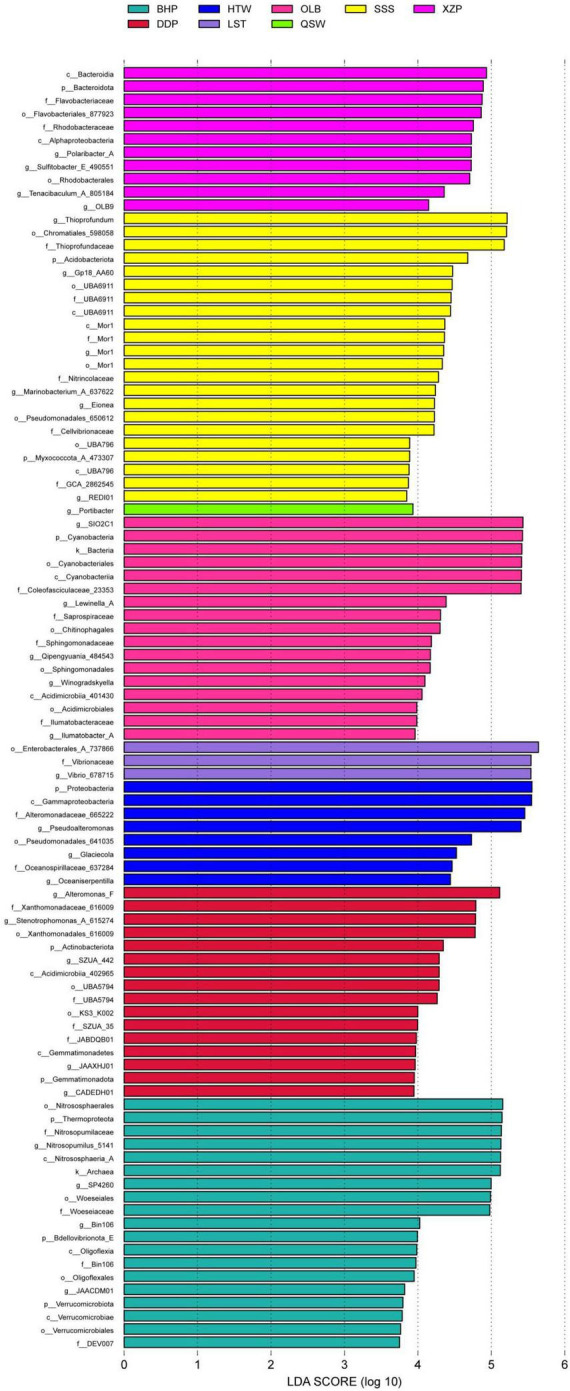
Analysis of characteristic species in different sites.

The analysis results of characteristic microbial species at different latitudes and longitudes are shown in Figure 5. The results showed that there were 97 distinct species groups in different latitudes and longitudes. There were up to 22 species with significant differences in SSS, and the most affected species were Thioprofundum. While the QSW had only one characteristic species (i.e., Portibacter). The microbial species of XZP mainly include 11 characteristic species such as Bacteroidia, Bacteroidota, Flavobacteriaceae, and so on. In addition, the species groups of other locations were significantly different as follows. The microbial species in XZP mainly included 11 species such as Bacteroidia, Bacteroidota, Flavobacteriaceae, and so on. There are a total of 17 characteristic species in OLB, including SIO2C1, and there are 8 species in HTW, such as Proteobacteria. There were 16 species in DDP such as Alteromonas_F, Xanthomonadaceae_616009, Stenotrophomonas_A_615274 and so on. There were 19 species in DDP, such as Nitrososphaerales, Thermoprpteota, Nitrosopumilaceae and so on.

### 3.3 Function prediction of sediment microbial communities at different sites

**FIGURE 6:**
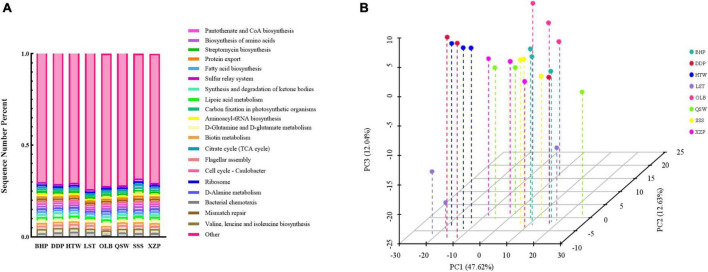
**(A)** KEGG path prediction consists of a bar graph. **(B)** Principal component analysis (PCA).

Functional prediction analysis of sediment microbial communities at different latitudes and longitudes was conducted, and the results are shown in Figure 6. According to the pathway prediction results of the KEGG database (Figure 6A), the microorganisms at the measured sites all had the following functions, namely, pantothenate and CoA biosynthesis, biosynthesis of amino acids, streptomycin biosynthesis, protein export, and fatty acid biosynthesis, etc. Among the top 20 functions, the biotin metabolism and valine, leucine, and isoleucine biosynthesis had the highest proportion. In the bargain, according to the PCA analysis of the KEGG database pathway, the contribution values of PC1 and PC2 were 47.62 and 12.63%, respectively, and the points in each group were not dispersed, indicating a small gap between samples, that is, there was no significant difference in the function of sediment microbial communities at different latitude and longitude (Figure 6B).

### 3.4 Relationship between latitude and longitude and water quality

**FIGURE 7:**
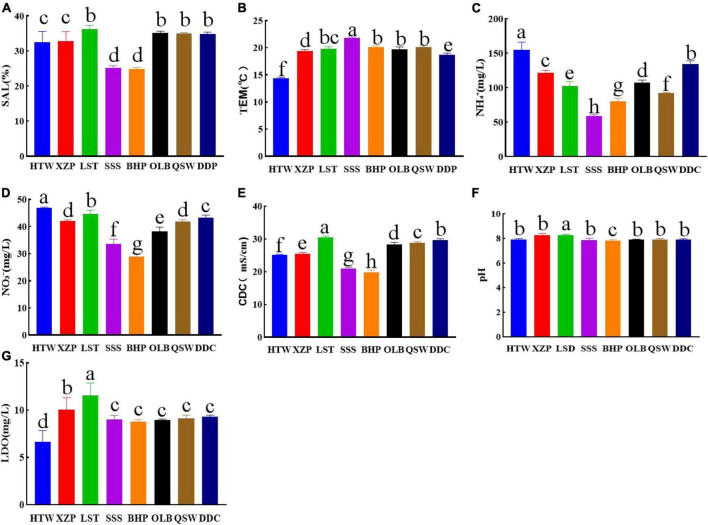
Different water quality factors include **(A)** SAL (Salinity). **(B)** TEM (Temperature). **(C)** NH_4_^+^. **(D)** NO_3_^–^. **(E)** CDC (Conductivity). **(F)** pH. **(G)** LDO (Dissolved oxygen).

The water quality in the coastal area of Quanzhou Bay varies with the latitude and longitude. The analysis of NO_3_^–^, SAL, TEM, pH, NH_4_^+^, CDC, and LDO showed that there was no significant difference in SAL between adjacent latitudes and longitudes (Figure 7A). The TEM is stable in the range of 14.30–21.85°C (Figure 7B), which belongs to the normal temperature range of offshore water. However, it can be seen from the results that there are significant differences in NO_3_^–^ and NH_4_^+^. The significant differences of NO_3_^–^ and NH_4_^+^ between 24°83′ and 24°94′N decreased with the increasing latitude (Figures 7C, D). In addition, according to the results, the measured CDC values have a large range of variation. The lowest CDC of BHP is 19.82 mS/cm, while the highest CDC of LST is 30.45 mS/cm (Figure 7E). As shown in Figure 7F, the pH value of the coastal waters of Quanzhou Bay is stable between 7.8 and 8.3, indicating that the water is weakly alkaline. According to the measured LDO data, the LDO values of HTW, LST, and XZP are 6.64, 11.58, and 10.04 mg/L, respectively. While the LDO values of other sites are stable in the range of 8.80–9.32 mg/L. The LDO varied significantly between 118°72′–118°78′E and 24°69′–24°81′N (Figure 7G).

### 3.5 Relationship between microorganisms and water quality

**FIGURE 8:**
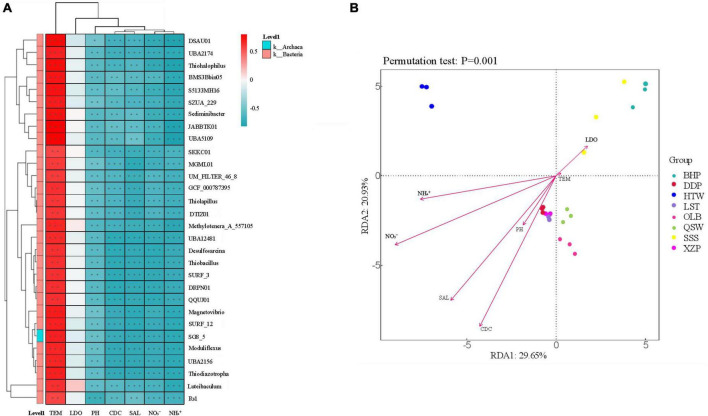
The relationship between sediment microbiota (at the genus level) and environmental factors. *R*-values (rank correlation) and *p*-values are obtained by calculation. *R*-values are shown in different colors in the figure, and if the *p*-value is less than 0.05, it is marked with *. The legend on the right is the color interval of different *R*-values. Meanwhile, the color bar on the left indicates the phylum classification to which the species belongs. *p < 0.05, **p < 0.01, ***p < 0.001. **(A)** Heat map of correlation between the environmental factors and generic level microorganisms. **(B)** RDA (Redundancy analysis).

In order to assess the relationship between environmental factors (i.e., water quality) and microbial community abundance, generic level correlation heat map analysis and RDA (Redundancy analysis) were performed, as shown in Figure 8. A total of 30 sediment microorganisms were closely related to 7 environmental factors depending on the latitude and longitude. 30 sediment microorganisms were positively correlated with TEM but negatively correlated with salinity, pH, CDC, NO_3_^–^, and NH_4_^+^. However, the correlation among these 30 sediment microbes and LDO was not strong (Figure 8A). On the side, according to the results of RDA analysis, the explanation rates of community structure differences on the two ranking axes were 29.65 and 20.93%, respectively, and the correlation between microbial communities and NO_3_^–^ was the largest, and TEM was the least. The microbial communities of BHP and SSS were positively correlated with LDO and TEM but negatively correlated with CDC, pH, SAL, NO_3_^–^, and NH_4_^+^. This is reversed in other locations (Figure 8B).

## 4 Discussion

Quanzhou Bay offshore area, as a typical transition zone between sea and land, is subjected to the triple coordinated pressure from the ocean, land, and atmosphere, and has obvious characteristics such as complex and diverse chemical, physical, and biological processes, coupled and changeable environmental factors, and sensitive and fragile ecological environment (Li et al., 2010; Chen et al., 2016; Yu et al., 2016; Bo et al., 2017; Wang et al., 2022). It is also a semi-enclosed bay located on the southeast coast of Fujian Province. The increasingly serious environmental pollution has brought great pressure to the marine ecological environment of Quanzhou Bay (Chen et al., 2016, 2018; Oberbeckmann and Labrenz, 2020). First of all, the sampling time of this study was spring. According to the data, the normal value of LDO in spring in the coastal waters of Quanzhou should be 6.37–8.24 mg/L, but the actual value of LDO measured was between 6.64 and 11.58 mg/L, which may be due to the proximity of the coastal water to urban and industrial waste water, the harmful substances reduce the oxygen consumption rate of activated sludge, resulting in the increase of LDO (Chen et al., 2018). Or because of the inflow of wastewater containing strong oxidants, resulting in the oxidation of bacterial cell material, preventing their normal cell metabolism and even causing bacterial death, reducing the microbial demand for oxygen, and ultimately increasing the content of LDO in the water (Phyu et al., 2024). From the increase of NO_3_^–^ and NH_4_^+^ contents in the water quality of coastal water, it can also be seen that the marine pollution caused by sewage discharge is aggravating (Akarsu et al., 2020). Secondly, the sediments in this study were collected from different sites, and it can be seen from the results that the microbial species, species classification, and structure of different sampling sites have significant changes (Nacke et al., 2011). These changes show that the environment of the coastal area of Quanzhou Bay is diverse (Chen et al., 2016; Yu et al., 2016). Although these patterns of change are disorganized as a whole, each location has its own special species, and these special species can be used as potential markers for environmental prediction (Chi et al., 2024). In addition, this study also determined the relationship between sediment microorganisms and water quality, whether positive or negative correlation, some specific microorganisms will depend on water quality to survive, so it can be used as an indicator to evaluate water quality detection. Finally, from the sediment microbial data and water quality index measured in this study, the current environmental status of the coastal area of Quanzhou Bay is still not optimistic, and it is urgent to protect it (Yu et al., 2008, 2016; Wang et al., 2022). The results of this experiment provide a scientific basis for protecting the environment of the coastal water of Quanzhou Bay.

## 5 Conclusion

Based on the complex and changeable environment of offshore water, this study carried out a study on environmental microorganisms and water quality within 150 km of offshore coastline of Quanzhou Bay. Our results show that there are irregular changes in the diversity of environmental microorganisms in the eight sites selected, including the relative abundance of microorganisms, and microbial composition, structure, and function. In addition, the water quality index also showed a disorderly change at different points, from this point of view, the results of our study exactly confirm the fact that the offshore environment is complex and changeable. In short, it can be confirmed by the research results that in recent years, the ecological environment risk of coastal water has increased due to the influence of human activities. The key data in this study, such as the characteristic microorganisms at each sampling point and the obvious changes in water quality at different sampling points, can be used as potential indicators to detect whether the offshore environment is polluted.

## Data availability statement

The data presented in the study are deposited in the NCBI repository. Available at: http://www.ncbi.nlm.nih.gov/bioproject/1129504, accession number PRJNA1129504.

## Author contributions

SD: Methodology, Formal analysis, Writing – original draft, Writing – review and editing. JC: Writing – review and editing. WZ: Conceptualization, Formal analysis, Writing – review and editing. SJ: Conceptualization, Writing – review and editing. YC: Data curation, Methodology, Investigation, Visualization, Writing – original draft, Writing – review and editing.

## Footnotes

1
https://www.bioincloud.tech
